# The use of topical corticosteroides in the treatment of oral lichen planus in Spain: A national survey

**DOI:** 10.4317/medoral.21435

**Published:** 2017-02-04

**Authors:** Laura Piñas, Abel García-García, Mario Pérez-Sayáns, Ricardo Suárez-Fernández, Mohammad-Hamdan Alkhraisat, Eduardo Anitua

**Affiliations:** 1 DDS, MPhil. MD, DDS, PhD. Private practice in oral implantology, Eduardo Anitua Foundation, Vitoria, Spain; 2 MD, PhD. DDS, PhD. Oral Medicine, Oral Surgery and Implantology Unit. Faculty of Medicine and Dentistry. Instituto de Investigación Sanitaria de Santiago (IDIS), Santiago de Compostela, Spain; 3 MD, PhD. Dermatology. Dermatology service General Universitary Hospital Gregorio Marañón, Madrid; 4 DDS, MSc, PhD, EU PhD. Clinical scientist. Eduardo Anitua Foundation, Vitoria, Spain

## Abstract

**Background:**

Explore the treatment of oral lichen planus with topical corticosteroids by the healthcare professionals in Spain.

**Material and Methods:**

A questionnaire targeted health professionals who treat OLP, in particular maxillofacial surgeons, dermatologist and dentist. The dissemination of the questionnaires was conducted through professional associations and dental and medical societies. The questionnaire was previously evaluated by means of a cognitive pre-test procedure to ensure that the questions were opportune and appropriate, understandable and acceptable among the professionals.

**Results:**

Of the 890 questionnaires sent a total of 190 questionnaires were answered by 90 dentists, 60 dermatol gists and 40 by maxillofacial surgeons. The most frequent treatment was 0.1% triamcinolone acetonide in orobase 3 times a day. The effectiveness of the topical corticosteroid treatment was 6.68 (SD= 2.26) in a scale of 1 to 10. The 30% of the dentists and 10.49% of maxillofacial surgeons combined treatment with other drugs. The most frequent one (80%) was nystatin (100,000 IU per millimetre). Dermatologists did not use other treatments in combination with corticosteroids.

**Conclusions:**

There is a need for national guidelines in treatment for oral lichen planus (treatment criteria, drug, dose, treatment time and method of application of corticosteroid) that can be applied by all professionals who treat this disease.

** Key words:**Oral lichen planus, topical corticosteroids, triamcinolone acetonide, questionnaire.

## Introduction

The term lichen planus (LP) was initially introduced by Erasmus Wilson in 1869 to redefine the condition that had been previously named leichen ruber by Hebra (1).

The first variant of LP was reported by Kaposi in 1892 and the first description of the peculiar striae was made by Louis Frederick Wickham (2,3). The histological findings were elaborated by Darier in 1909 (4).

Lichen planus (LP) is a chronic autoimmune mucocutaneous condition which most commonly affects middleaged adults of both sexes with a slight predominance in women (ratio 1.4:1). LP can affect the oral mucosa, skin, genital mucosa, scalp and nails. The prevalence of oral lichen planus (OLP) in the general population ranges between 0.5-2.6% with variations between different countries: 0.5% in Japanese population, 1.9% in Swedish population, 2.6% in the Indian population and 0.38% in Malaysian population (5-9). In Spain, the prevalence of LP was 0.2-2% (10).

Clinically we can observe four forms of OLP: reticular form, atrophic-erosive form, plaque form and papular form (5,11). Lesions are typically bilateral and burning sensation and sometimes pain usually accompany the erosive type lesion (12). Several factors have been proposed for the aetiology of OLP including: genetic factors, dental materials, drugs, infectious agents, bacterial and viral infections, autoimmunity (autoimmune diseases), immunodeficiency, food allergies, stress, habits, trauma, diabetes, malignant neoplasm and bowel diseases (1,11-13). The pathogenesis of OLP includes antigen-specific and non-specific mechanism. Antigenspecific mechanism in OLP include antigen presentation by basal keratinocytes and antigen-specific keratinocyte killing by CD8+ cytotoxic T cells. Non-specific mechanism include mast cell degranulation and matrix metalloproteinase activation in OLP lesions (13,14).

Several treatment regimens have been proposed to improve management of symptomatic OLP: corticosteroids, retinoids, immunosupressors (cyclosporine, levamisole and azithioprine), antifungal agents and psoralen and ultraviolet A radiation (PUVA) therapy (15).

Topical treatment is generally preferred as it has fewer adverse effects. Systemic agents was indicated if lesions are disseminated and mainly involve the skin, mucosa or when topical therapies are not effective.

Topical corticosteroids are the most useful agents for the treatment of OLP but there is a lack of adequate studies determining their efficacy and optimal dose, duration of treatment and type of formulation (15-19). The aim of this article is to explore the different topical corticosteroids for the treatment OLP and compare treatment regimen according to the healthcare professionals in Spain.

A questionnaire has been developed and has been sent to healthcare professionals that treat OLP in Spain.

## Material and Methods

In order to know the type of treatment used with topical corticosteroids for the treatment of oral lichen planus a questionnaire was sent by mail (web link) to healthcare professionals in Spain.

- Measurement tool

A questionnaire targeted health professionals who treat

OLP, and in particular maxillofacial surgeons, dermatologist and dentist. The dissemination of the questionnaires was conducted through professional associations and dental and medical societies. Anonymity in completing the questionnaire was sought in all cases. The link sent redirect participants to a website where the questionnaire was filled anonymously and later sent to a database.

The questionnaire was previously evaluated by means of a cognitive pre-test procedure to ensure that the questions were opportune and appropriate, understandable and acceptable among the professionals. This pilot survey was targeted to 10 dental professionals selected due to their accessibility and proximity to the investigational team.

- Statistical analysis

The medical professional was the statistical unit of analysis. Frequency analysis was performed for categorical variables and mean ± standard deviation was calculated for continuous variable. The results were analyzed using SPSS v15.0 for Windows statistical software package (SPSS Inc., Chicago, IL, USA).

## Results

- Participants

Of the 890 questionnaires sent a total of 190 (21.34%) questionnaires were answered and processed. Ninety questionnaires were answered by dentists, 60 by dermatologists and 40 by maxillofacial surgeons. The 10% of the professionals have an experience of 1-5 years, 39.47% of 6-10 years, 10.1% of 11-15 years, 10% of 16- 20 years, 8.42% of 21-25 years, 7.89% of 26-30 years and 13.15% of more than 30 years. The 90% of respondents realize the diagnosis of OLP by clinical findings and biopsy. The remaining 10% used only the clinical findings. The most common treated form of OLP was the atrophic-erosive form (65%) followed by the reticular form (22.12%) and the less treated form was the plaque form 12,88%).

- Topical corticosteroids

Type: The most frequent topical corticosteroids was triamcinolone acetonide (71.57%), followed by fluamcinolone acetonide (15.26%) and clobetasol propionate (12.10%). The data according to the medical professional were as the following: triamcinolone acetonide was used by in 78% of the dentists, fluamcinolone acetonide by 14.23% and clobetasol propionate by 7.77% . Meanwhile, triamcinolone acetonide was used by e 60% of the dermatologists, fluamcinolone acetonide by 20% and clobetasol propionate by 20%. Triamcinolone acetonide was used by 75% of the maxillofacial surgeons, fluamcinolone acetonide by 15% and clobetasol propionate by 10%.

Mode of application: The most common mode was paste (62,63%). This mode of application was used by 55.56 of the dentists, 66.66% of the dermatologists and 75% of the maxillofacial surgeons. The solution form was the second mode of application (35.26%). This mode of application was used by 44.44% of the dentists, 28.34% of the dermatologist and 25% of the maxillofacial surgeons. The less frequent mode of application was oily ointment (2.11%). This form of application was only used by 5% of the dermatologists.

Frequency and concentration: The daily regimen mostly employed was 3 times a day (46.60%) follow by two times a day (21.4%) and one single application (20%). Four times a day was the least frequent (12%). The daily dose prescribed by the medical professionals is detailed in  Table 1 and the concentration of each corticosteroid in  Table 2. The most frequent concentrations were 0.1% triamcinolone actonide, 0.05% fluamcinolone acetonide and 0.05% clobetasol propionate.  Table 3 shows the results of combining the type of corticosteroid, dosage, mode of application and daily regimen. The most frequent combination was 0.1% triamcinolone acetonide paste 3 times a day. The time for which this treatment had been maintained was variable. The 10% of professionals kept it for 15 days, the 15% for one month, the 35% for 1.5 months and the remaining 40% for two months.

**Table 1:**
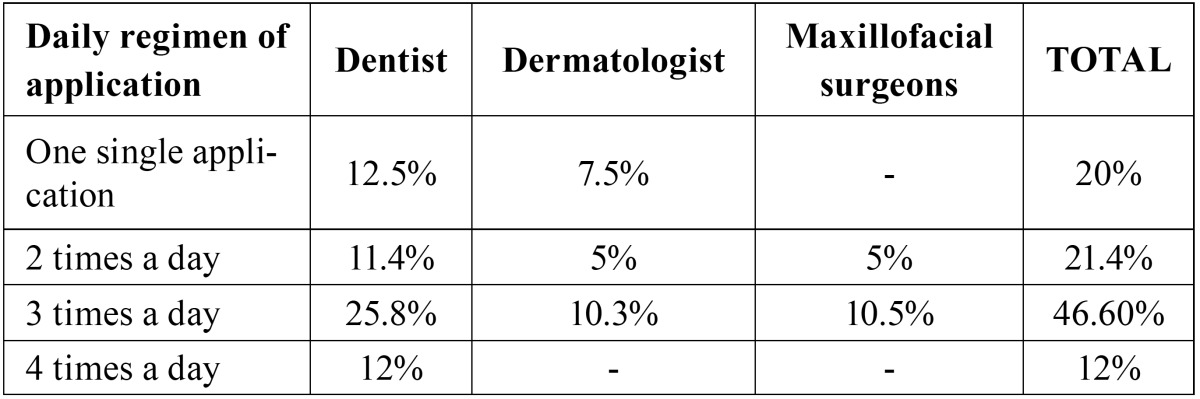
Daily regimen of application of corticosteroid by medical specialty.

**Table 2:**
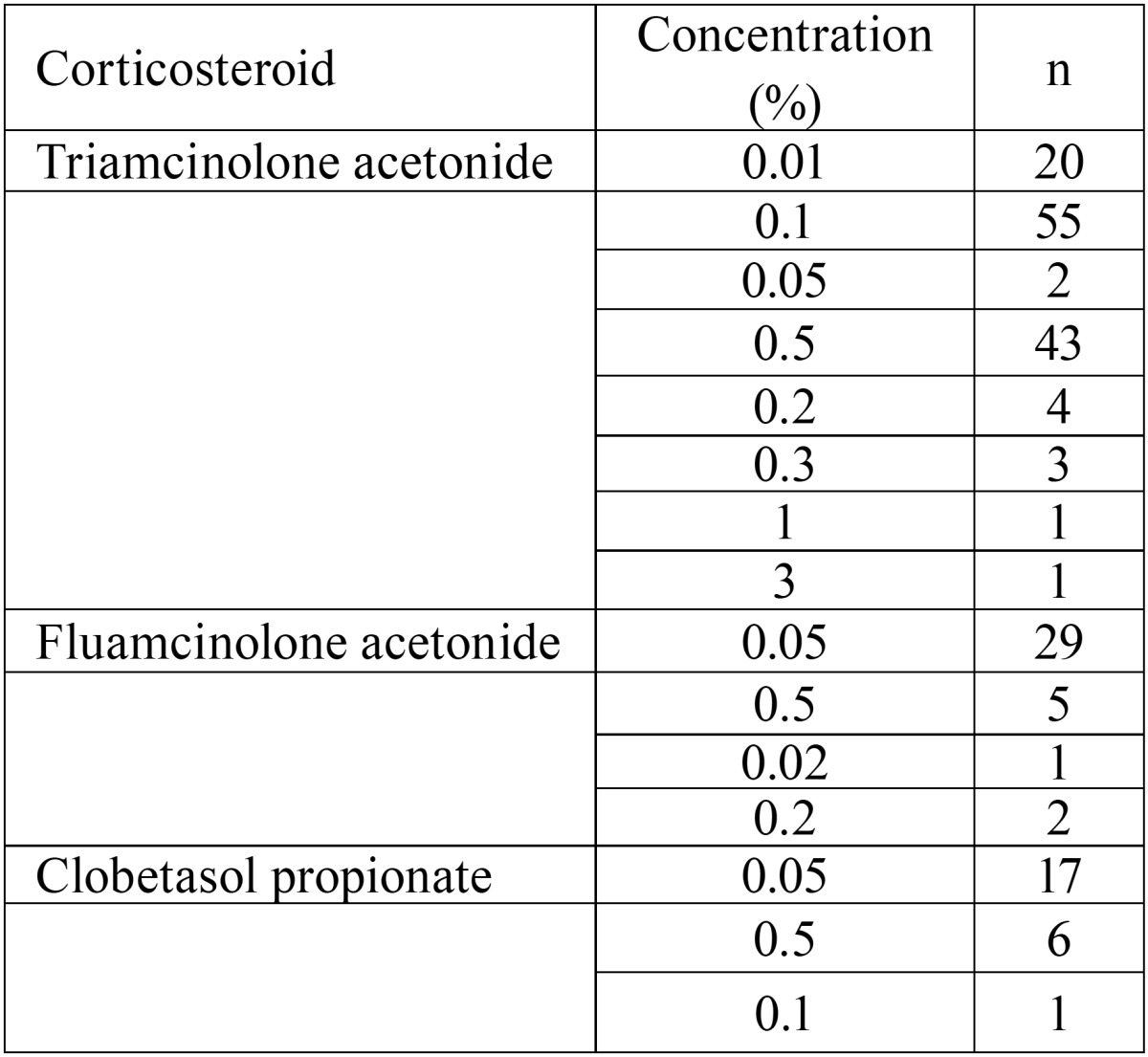
Corticosteroid and concentration.

**Table 3:**
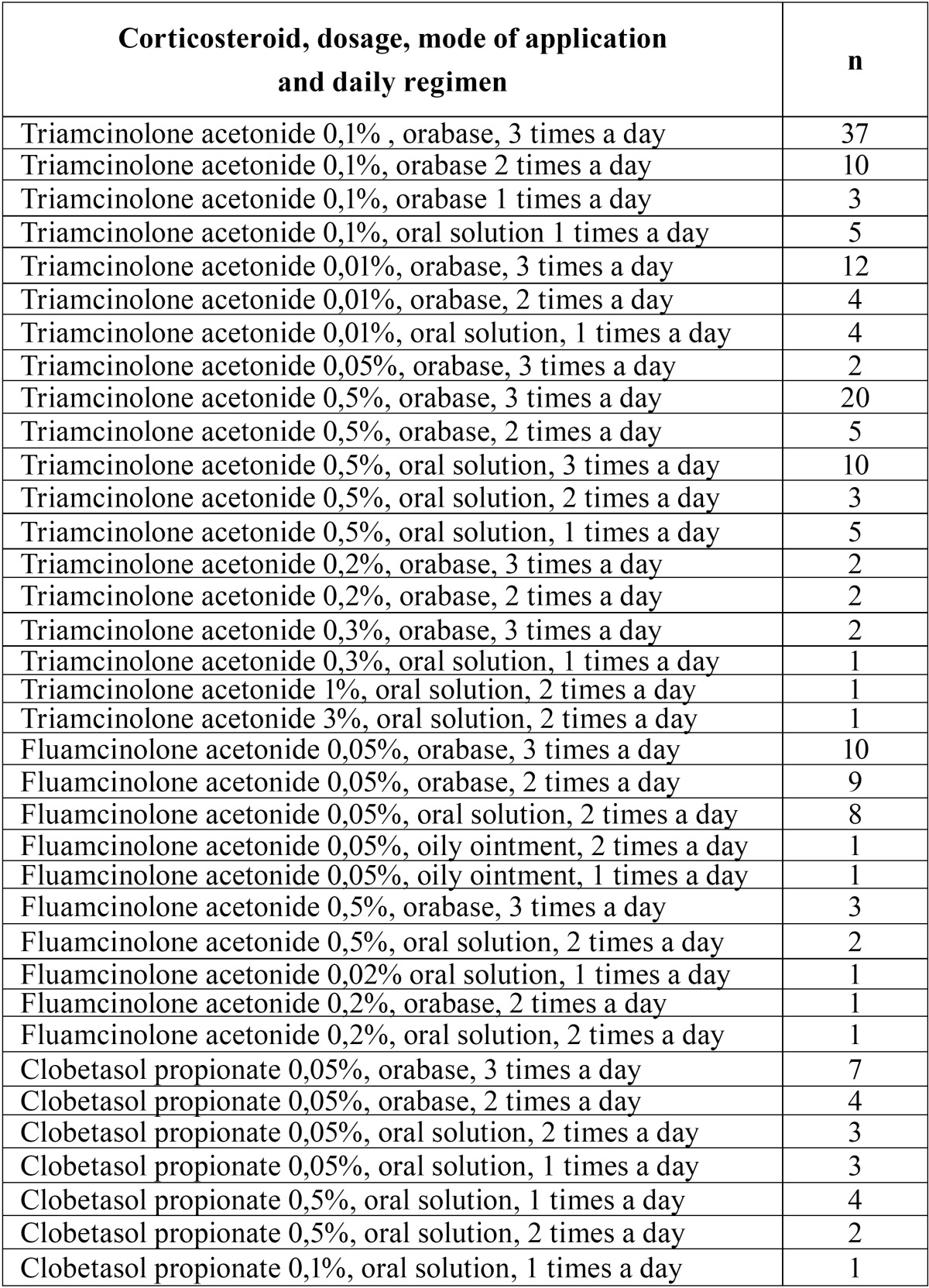
Corticosteroid, dosage, mode of application and daily regimen.

**Figure 1:**
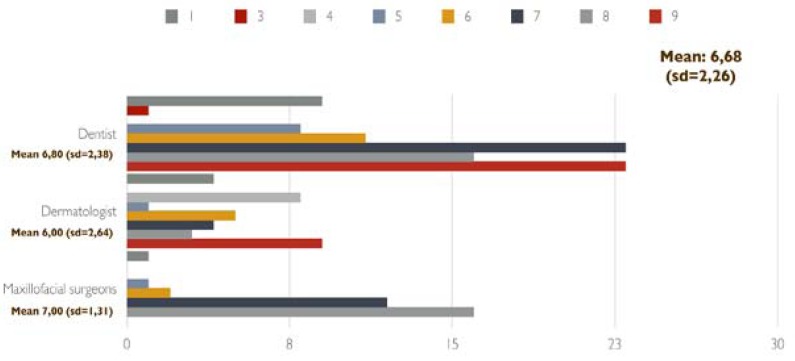
Effectiveness of the corticosteroid treatment in a score of 1 to ten points by medical specialty.

Efficacy: The effectiveness of the corticosteroid treatment was scored in a scale of 1 to 10 (1 was no effectiveness and 10 the most effective). The mean value of the evaluations were 6.68 (SD= 2.26). The evaluations by medical specialty are shown in figure 1. The effectiveness of triamcinolone acetonide 0.1% paste, 3 times a day (the most frequent combination), was rated 7.1 (SD=1.18).

- Other treatments

The participants in the questionnaires were asked if they added other medicaments to the usual treatment with topical corticosteroids. The answer was Yes by 40.49%, being 30% dentists and 10.49% maxillofacial surgeons. All dermatologists did not use other treatments in combination with corticosteroids. The most frequent medicament (80%) was nystatin (100,000 IU per millimetre), followed by retinoid acid (15%) and hyaluronic acid (5%). Dentists only added nystatin to the treatment of OLP.

## Discussion

A review of the scientific literature has revealed only one study similar to our own, exploring the treatment of oral lichen planus in Spain. In the study of Lóper-Jornet *et al.* (20) report a necessity of guidelines to treat oral lichen planus. The most frequent treatment reported, according to Lóper-Jornet *et al.* (20), was topical corticoids (25%), followed by mouthwashes such as triclosan (20%) or clorhexidine (20%). Systemic corticoids were used by 6% of respondents, immunosuppressors by 2.6% and other treatments by 1.5%. (20) If we unificate the employment of triclosan and clorhexidine in a single group we can observe as there is a majority (40%) of professionals who treat the oral lichen planus with different drugs to corticosteroids, these being the first line of action according to the majority of the consulted studies (10-19).

The study methodology (mailed questionnaires) has been widely used, though the response rates elicited with this approach are highly variable. López-Jornet *et al.* (20) obtained a 74% response rate, Payne (21) obtained a 71% response rate in a study centered on dentists, while Cowan *et al.* (22) recorded a 67% response rate. In contrast, Warnakulasuriya and Johnson documented a rate of only 16% (23). Our response rate was 21.34%, similar to Warnakulasuriya and Johnson (23). This low response rate makes caution necessary in drawing conclusions from the results obtained, and precludes extrapolation of the findings to the global population of health professionals who treat OLP. However, the data in this work represent, to the best of our knowledge, the first insight into how OLP is treated with topic corticosteroids by the Spanish health professionals.

Corticosteroids have been found to be the most predictable and successful agents in treatment of oral lichen planus. Topical application is the treatment of choice, as it can be effectively delivered to the lesion with minimal potential for systemic side effects (15-18). The efficacy of corticosteroids for treatment of OLP is mainly attributed to its anti-inflammatory and immunosuppressive actions (18). Many trials and formulations of corticosteroids have been used in the treatment of OLP. In the Cochrane review of placebo-controlled randomized clinical trials of treatments used for symptomatic OLP, there have been no trials comparing topical steroids with placebo. There are many trials comparing different steroids with different alternative treatments (retinoids, immunosupressors -cyclosporine, levamisole and azithioprine-, and ultraviolet A radiation (PUVA) therapy) and there is no evidence that one steroid treatment is better or worse than another (19).

In our study the most frequent treatment option was triamcinolone acetonide 0.1%, orabase, 3 times a day. This treatment is the less employed by the most of the clinical trials published in relation to corticosteroids treatment of OLP (19). Yoke *et al.* (24) compare topical triamcinolone acetonide 0.1% versus topical cyclosporine solution (100 mg/ml) in a randomized controlled trial. Clinical response, pain, burning sensation, area of reticulation, erythema, and ulceration at week 4 were all worse in patients receiving cyclosporine than in those receiving steroid, however the differences were not statistically significant. There was a statistically significant reduction in the mean area of ulceration in the triamcinolone group. Laeijendecker (25) *et al.* have compared topical treatment of OLP with triamcinolone acetonide 0.1% or tacrolimus 0.1%. A better initial therapeutic response was assoicated with tacrolimus 0.1%, however relapses occurred frequently within 3-9 weeks of the cessation of treatment. Triamcinolone acetonide 0.1% in paste has been compared with betamethasone oral mini-pulse (OMP) therapy and the results indicated that topical triamcinolone acetonide was equally effective (26). The same results has been obtained when triamcinolone acetonide 0.1% has been compared to prednisolone 5 mg mucoadhesive tablet (27).

In fact, there is no clinical trials that have compared the effectiveness of different corticosteroids in the treatment of OLP. However, triamcinolone acetonide was considered in several studies as the treatment of choice with good results. In our study, the average rating for the effectiveness of treatment using triamcinolone acetonide 0.1%, orabase, 3 times a day was 7.1 (SD=1.18), the overall scoring of the effectiveness of topical corticosteroids was 6.68 (SD= 2.26).

Finally, it is important to highlight the need for national guidelines of treatment for oral lichen planus that can be applied by all professionals who treat this disease. Thus, the treatment criteria are much clearer and similar patterns of drug, dose, treatment time and method of application of corticosteroid can be followed. The low response rate of the questionnaires limits the extrapolation of the study outcomes to the management of OLP with topical corticosteroids in Spain. Moreover, there is a need for a scientific evidence of the effectiveness of topical corticosteroids in the symptomatic treatment of oral lichen planus.
